# CACLA-Based Trajectory Tracking Guidance for RLV in Terminal Area Energy Management Phase

**DOI:** 10.3390/s21155062

**Published:** 2021-07-26

**Authors:** Xuejing Lan, Zhifeng Tan, Tao Zou, Wenbiao Xu

**Affiliations:** 1 School of Mechanical and Electrical Engineering, Guangzhou University, Guangzhou 510006, China; lanxj@gzhu.edu.cn (X.L.); tzf710647@163.com (Z.T.); 2 Guangdong Province Institute of Metrology, Guangzhou 510450, China; xwb8911@scm.com.cn

**Keywords:** RLV, guidance, TAEM, CACLA, TD learning

## Abstract

This paper focuses on the trajectory tracking guidance problem for the Terminal Area Energy Management (TAEM) phase of the Reusable Launch Vehicle (RLV). Considering the continuous state and action space of this guidance problem, the Continuous Actor–Critic Learning Automata (CACLA) is applied to construct the guidance strategy of RLV. Two three-layer neuron networks are used to model the critic and actor of CACLA, respectively. The weight vectors of the critic are updated by the model-free Temporal Difference (TD) learning algorithm, which is improved by eligibility trace and momentum factor. The weight vectors of the actor are updated based on the sign of TD error, and a Gauss exploration is carried out in the actor. Finally, a Monte Carlo simulation and a comparison simulation are performed to show the effectiveness of the CACLA-based guidance strategy.

## 1. Introduction

Advanced Reusable Launch Vehicle (RLV) is a space vehicle that can transport people or payloads into a predetermined orbit and can be reused many times [1,2]. RLV highly integrates and develops aerospace technology and aeronautics technology. It is the inevitable trend of the development of the space transportation systems and has important military and civil values [3]. Therefore, many countries have researched RLV to reduce the cost of future space transportation [4,5].

The main return modes of RLV are parachute vertical descent scheme, thrust reversing vertical landing scheme, and gliding flight horizontal landing scheme. In this paper, RLV uses the gliding flight horizontal landing scheme, which has a long deceleration time, less overload, and a wider re-entry corridor. Because RLV has no engine thrust to re-fly during its return, it is necessary to strictly manage the remaining energy of RLV to ensure its safe horizontal landing. The return process of RLV includes the initial re-entry stage, the Terminal Area Energy Management (TAEM) stage and the automatic landing stage [6,7,8]. In the initial reentry phase, the atmosphere is thin, and the trajectory control ability of RLV is weak [9,10,11]. In the automatic landing phase, RLV is very close to the ground, and the adjustable range and remaining time of the trajectory are very limited [12,13]. Therefore, TAEM is the most important phase for the return landing mission [14,15].

The energy change of RLV in TAEM is closely related to the trajectory shape. Thus, the RLV must track the reference trajectory to ensure the safe flight and accurate landing [16,17]. However, the complex flight environment and mission requirements pose challenges to the TAEM guidance system [18,19,20]. In the research of guidance algorithms, the mature guidance method is to use a small perturbation approximation method or feedback linearization theory to obtain the linear model of RLV. Then, the guidance law is designed based on robust control theory or Linear Quadratic Regulator (LQR) to track the reference trajectory [21,22,23]. The performance of these methods has a tight relationship with the accuracy of RLV modeling. At present, the research on such guidance methods is focused on reducing the impact of interference and uncertainty on the system. The guidance algorithm directly oriented to the nonlinear model of RLV includes sliding mode theory, fuzzy theory, and adaptive theory [24,25]. However, these methods still have some difficulties in engineering implementation and need to be further studied. On the other hand, under the influence of a complex flight environment, RLV may deviate from the preset flight trajectory seriously and can not return to the preset flight trajectory. Thus, the online autonomous reconstruction of reference trajectory is an effective way to improve the reliability of RLV [26,27,28]. Then, the guidance law based on the preset reference trajectory cannot apply to the tracking task of the newly reconstructed reference trajectory. Therefore, this paper intends to study an intelligent guidance technology to achieve the adaptive tracking of the reconstructed reference trajectory.

Reinforcement Learning (RL), as a kind of algorithm in the machine learning field, follows the idea of human learning through environmental feedback, with the aim to solve the guidance problem of RLV in the complex flight environment. To the best of our knowledge, the research on the combination of RL and traditional TAEM guidance is still rare. At present, traditional RL algorithms mainly solve problems with finite discrete action and state space [29,30,31]. However, many practical problems (such as the guidance problem discussed in this paper) have continuous state and action space, which makes learning a good strategy more complex [32,33,34,35]. Therefore, scholars have done much research on RL in the continuous domain [36,37,38,39]. The actor–critic algorithm is an effective method to deal with the problem of the “curse of dimension” based on the application of function approximation technology [40,41,42]. In addition, an improved actor–critic algorithm called Continuous Actor–Critic Learning Automata (CACLA) is developed and performs well in the scene with continuous action and state space, such as LTE-A cellular networks and robotic control tasks [43,44,45].

To improve the intelligence and adaptiveness of RLV, in this paper, we will use CACLA to construct a trajectory tracking guidance strategy for the TAEM phase of RLV. The Markov Decision Process (MDP) of the guidance problem is modeled based on the trajectory tracking errors and the guidance command increments. The critic and actor of CACLA are modeled by two three-layer neuron networks, respectively. The online weight learning process is realized by an improved model-free Temporal Difference (TD) learning algorithm. Then, the guidance commands of RLV are obtained based on a Gauss exploration in the actor. Compared with the existing research on guidance strategies, the main contributions of this paper are as follows:

(i) An intelligent trajectory tracking guidance strategy is proposed based on CACLA for RLV in terminal area energy management phase.
(ii) The guidance strategy is a data-based guidance method with the ability to learn online, with no need to know the accurate system model.
(iii) The guidance strategy has good adaptability and robustness, and can be used to track the reconstructed reference trajectory.

## 2. Problem Formulation

### 2.1. Dynamics of RLV

The RLV to be studied in this paper uses the gliding flight horizontal landing scheme. There is no engine thrust during the return process, and the gliding maneuvering return depends on the aerodynamic force generated by the movement of RLV in the atmosphere. This kind of return mode can make a proper orbit manoeuvre by controlling the direction of lift, thus creating good conditions for horizontal landing on the runway of the landing site. In this paper, it is assumed that the earth is flat and non-rotating in TAEM. Thus, the dynamic model of RLV is established as follows:

(1) $$\dot{v}=-\frac{D}{m}-g\sin\gamma ,$$

(2) $$\dot{\gamma }=\frac{L\cos\sigma }{mv}-\frac{g\cos\gamma }{v},$$

(3) $$\dot{\chi }=\frac{L\sin\sigma }{mv\cos\gamma },$$

(4) $$\dot{h}=v\sin\gamma ,$$

(5) $$\dot{x}=v\cos\gamma \cos\chi ,$$

(6) $$\dot{y}=v\cos\gamma \sin\chi ,$$

where the states are calculated based on a landing coordinate system. *v*, *h*, and γ represent the velocity, altitude, and flight path angle of RLV, respectively. *x* and *y* represent the longitudinal and lateral positions of RLV in the landing coordinate system. χ is the heading angle relative to the runway centerline. *m* represents the mass of vehicle, and *g* represents the gravitational acceleration. The bank angle of RLV is denoted by σ. In addition, *L* and *D* denote the aerodynamic lift force and drag force, respectively, as follows:

(7) $$L=qSC_{L},$$

(8) $$D=qSC_{D},$$

where *S* is the reference area of vehicle, and *q* is the dynamic pressure. $C_{L}$ ($C_{D}$) denotes the aerodynamic lift coefficient (drag coefficient), which can be determined by the angle-of-attack α and the Mach number *M* with a two-dimensional table look-up.

The total state vector of RLV is concluded as

(9) $$X={\left[v,\gamma ,\chi ,h,x,y\right]}^{T}.$$

The nominal reference trajectory can be obtained by off-line trajectory planning or online trajectory planning algorithms. The tracking error vector is defined according to the current state vector *X* and the state vector of reference trajectory $X_{r}$:

(10) $$\Delta X=X-X_{r}.$$

At the end of the TAEM, the RLV must achieve the desired Approach and Landing Interface (ALI) states $X_{ALI}$ to ensure the safety of the automatic landing phase. In view of the trajectory tracking guidance method, the TAEM terminal states can meet the ALI constraints by accurate tracking the reference trajectory with the tracking error satisfies

(11) $$\left|\Delta X\right|\leq \varepsilon_{X},$$

where $\varepsilon_{X}$ is the boundary of trajectory tracking error. Although there is no strict restriction on the terminal flight path angle, in order to ensure the high-precision tracking of the reference trajectory, the flight path angle still needs to be considered in the guidance strategy.

### 2.2. Markov Decision Processes

The trajectory tracking problem of RLV should first be modeled as a Markov Decision Process (MDP) to enable the work of CACLA . The state of MDP *s* is defined by the trajectory tracking errors:

(12) s=ΔX,

The action *a* is defined based on the guidance command increments:

(13) $$a={\left[\Delta \alpha ,\Delta \sigma \right]}^{T}.$$

where $\Delta \alpha =\alpha -\alpha_{r}$ is the increment of current angle of attack α relative to the reference one $\alpha_{r}$. $\Delta \sigma =\sigma -\sigma_{r}$ is the increment of current bank angle σ relative to the reference bank angle $\sigma_{r}$.

The immediate reward *r* is defined by

(14) $$r=s^{T}Ms+a^{T}Ga,$$

where $M^{T}=M>0$ and $G^{T}=G>0$ are square matrices. The smaller the error between the current state and the reference state, the smaller the immediate reward. V(s) is the state value function represents the expected accumulative total rewards from the state *s*. The task of trajectory tracking guidance is to solve an optimal control strategy to minimize the state value function V(s).

## 3. CACLA-Based Guidance Strategy

### 3.1. CACLA Algorithm for Trajectory Tracking

CACLA is a reinforcement learning algorithm that can be effectively implemented in continuous state and action space. In this paper, we will use CACLA to learn a guidance law for RLV to track the reference trajectory. There are two modules in CACLA, named “critic” and “actor”. In this work, two three-layer neuron networks (NN) are used to realize the function approximation for the critic and actor, respectively. In the critic, there are 6 input layer neurons, *k* hidden layer neurons, and 1 output layer neuron. In the actor, there are 6 input layer neurons, *q* hidden layer neurons, and 2 output layer neurons. Then, the state value function is approximated as

(15) $$V\left(s\right)=\theta_{2}\cdot \phi \left(\theta_{1}s\right),$$

where $\theta_{1}\in R^{k\times 6}$ and $\theta_{2}\in R^{1\times k}$ are the weight vectors of NN in critic. ϕ(·) is the basic function defined as

(16) $$\phi \left(z\right)=\frac{1-\exp(-z)}{1+\exp(-z)}.$$

Moreover, the action function is approximated as

(17) $$A\left(s\right)=\psi_{2}\cdot \phi \left(\psi_{1}s\right),$$

where $\psi_{1}\in R^{q\times 6}$ and $\psi_{2}\in R^{2\times q}$ are the weight vectors of NN in actor. To enable the exploration in CACLA, a Gaussian distribution policy P(s,a) centered on A(s) is defined:

(18) $$P\left(s,a\right)=\frac{1}{\sqrt{2\pi }\mu }\exp(-\frac{{\left(a-A(s)\right)}^{2}}{2\mu^{2}}),$$

where π and μ are constant parameters. Thus, the action *a* is achieved according to this Gauss exploration.

From the definition in (13), we can further obtain the actual guidance commands α and σ based on the known reference commands:

(19) $${\left[\alpha ,\sigma \right]}^{T}=a+{\left[\alpha_{r},\sigma_{r}\right]}^{T},$$

When the guidance commands α and σ are applied to the dynamic model of RLV, the next total state vector of RLV can be obtained. Compared to the states and commands of the given reference trajectory, the next state of MDP s(t+1) and the immediate reward r(t) can be obtained. Then, the model free TD learning algorithm is used to update the weight vectors of critic.

(20) $$\theta_{1}\left(t+1\right)=\theta_{1}\left(t\right)+\Delta \theta_{1}\left(t+1\right),$$

(21) $$\theta_{2}\left(t+1\right)=\theta_{2}\left(t\right)+\Delta \theta_{2}\left(t+1\right),$$

(22) $$\Delta \theta_{1}\left(t+1\right)=\eta \Delta \theta_{1}\left(t\right)+\varsigma \left(1-\eta \right)e_{1}\left(t+1\right)\delta (t),$$

(23) $$\Delta \theta_{2}\left(t+1\right)=\eta \Delta \theta_{2}\left(t\right)+\varsigma \left(1-\eta \right)e_{2}\left(t+1\right)\delta (t),$$

where η is the momentum factor, and ς is the learning rate. $e_{1}\in R^{k\times 6}$ and $e_{2}\in R^{1\times k}$ are eligibility traces updated as follows:

(24) $$e_{1}\left(t+1\right)=\lambda \tau e_{1}\left(t\right)+\nabla_{\theta_{1}}V_{t}\left(s\left(t\right)\right),$$

(25) $$e_{2}\left(t+1\right)=\lambda \tau e_{2}\left(t\right)+\nabla_{\theta_{2}}V_{t}\left(s\left(t\right)\right),$$

where τ is the discount factor, and λ is a trace decay parameter. δ(t) is the TD error defined as

(26) $$\delta \left(t\right)=r\left(t\right)+\tau V_{t}\left(s(t+1)\right)-V_{t}\left(s(t)\right).$$

If the TD error δ(t)>0, the weight vector of actor will be updated by

(27) $$\psi_{1}\left(t+1\right)=\psi_{1}\left(t\right)+\beta (a\left(t\right)-A_{t}\left(s\left(t\right)\right)\nabla_{\psi_{1}}A_{t}\left(s(t)\right),$$

(28) $$\psi_{2}\left(t+1\right)=\psi_{2}\left(t\right)+\beta (a\left(t\right)-A_{t}\left(s\left(t\right)\right)\nabla_{\psi_{2}}A_{t}\left(s(t)\right),$$

where β is a learning rate. a(t) is the explored action, and $A_{t}\left(s\left(t\right)\right)$ is the output of the actor.

It can be seen that the actor update process is performed only when the TD error is positive. Therefore, the actor of CACLA is updated based on the sign of TD error, not on the value of TD error as other actor–critic learning methods do. Moreover, another difference from most other actor–critic learning methods is that CACLA directly update the actor by the error in the action space, not the error in the policy space.

### 3.2. Application of Guidance Strategy

**Figure 1:**
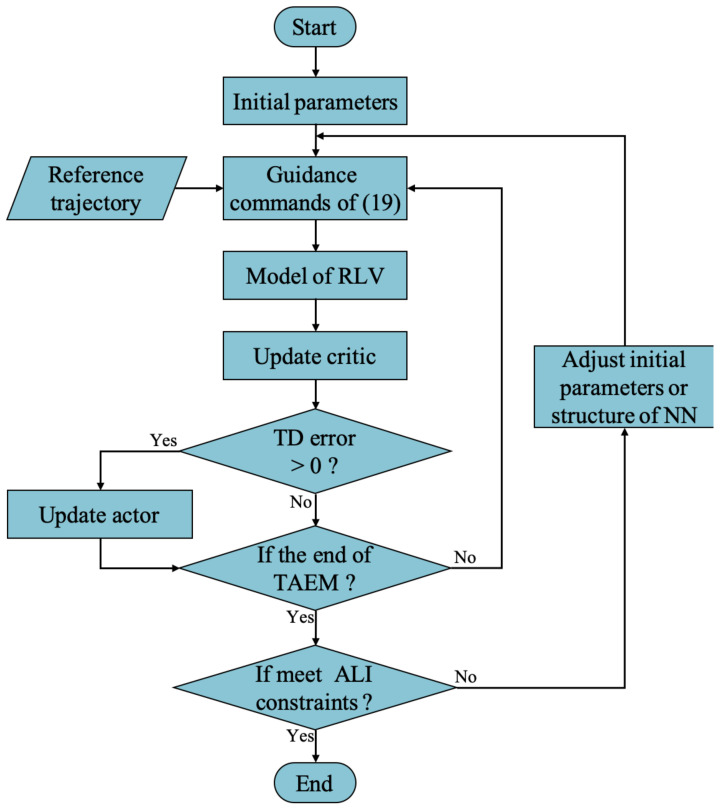
The flowchart of the off-line training of CACLA.

Due to the high cost of RLV, it is necessary to train the critic and actor offline before the guidance strategy is implemented in RLV. The flowchart of the off-line training of CACLA is shown in Figure 1. The updating procedure of critic and actor is performed at each step of TAEM, and is continued until RLV reaches the terminal ALI. At the end of TAEM if RLV has not met the ALI constraints, the TAEM guidance process will be performed again from the start of TAEM with adjusted initial parameters or structure of NN. For example, the latest updated weight vectors of the critic and the actor can be used as the initial values to achieve better guidance accuracy. When the off-line training of critic and actor achieves the required guidance accuracy, the weight vectors of critic and actor can be saved and be used in practical guidance missions.

**Figure 2:**
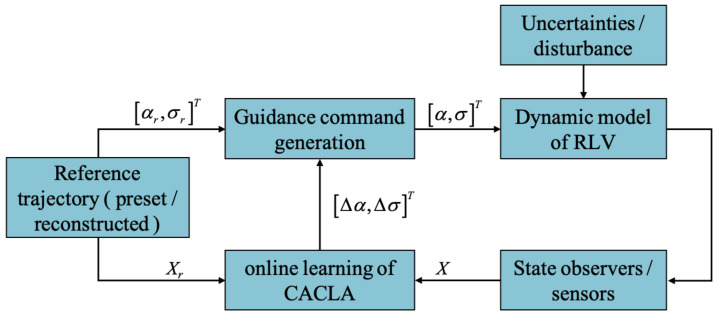
The framework of guidance system.

The online learning of CACLA is effective to ensure the adaptive tracking of the reference trajectory, which may be reconstructed online to improve the reliability of RLV. On the other hand, even if the reference trajectory is not reconstructed, the online learning of CACLA is also necessary and helpful to improve the intelligence level of the guidance system to cope with the impact of the complex environment. Figure 2 shows the framework of the CACLA-based guidance system. The online learning procedure of guidance strategy is the same as the offline training. The weight vectors of critic are updated by (20)–(23). The weight vectors of actor are updated by (27) and (28), but only when the TD error is positive. Based on the output of the actor and the reference commands, the actual guidance commands are obtained by (19) and applied to the dynamic model of RLV. In order to simulate the actual flight TAEM environment of RLV, uncertainties and disturbances are added to the flight process. In this paper, it is assumed that the guidance system can accurately know the states of RLV through sensors or observers. Therefore, the research on state perception error or observation error will not be discussed in detail.

## 4. Simulation Results

In this section, a Monte Carlo simulation and a comparison simulation are performed to evaluate the effectiveness of the proposed intelligent guidance strategy. In the TAEM phase, the velocity and altitude are initialized as v=900 m/s and h=28 km. The lateral ground track position is initialized as x=−10 km and y=−50 km. The flight path angle is initialized as γ=−8 deg, and the initial heading angle is set towards the ALI. The desired ALI conditions are defined as v<180 m/s, h=3±0.1 km, x=−21±0.3 km, y=0±0.1 km, and χ=0±5 deg. Although there is no strict restriction on the terminal flight path angle, to ensure the high precision tracking of the reference trajectory, the flight path angle still needs to be considered in the guidance strategy. Thus, the boundaries of trajectory tracking errors for each state of RLV are set as $\varepsilon_{v}=100$ m/s, $\varepsilon_{\gamma }=5$ deg, $\varepsilon_{\chi }=5$ deg, $\varepsilon_{h}=0.1$ km, $\varepsilon_{x}=0.3$ km, and $\varepsilon_{y}=0.1$ km. In addition, the guidance commands are subject to the bank-angle rate limit of 10 deg/s and the angle-of-attack rate limit of 10 deg/s in the simulation. The parameters of CACLA are set as η=0.2, ς=0.4, τ=0.2, λ=0.1, β=0.1, k=10, and q=8. The initial $\Delta \theta_{1}\left(0\right)$, $\Delta \theta_{2}\left(0\right)$, $e_{1}\left(0\right)$, and $e_{2}\left(0\right)$ are set as zero.

### 4.1. Monte Carlo Simulation

In the actual flight process, the deviations of the aerodynamic model and atmospheric density model inevitably exist due to modeling uncertainties or unknown disturbances. In order to evaluate the performance of the CACLA-based guidance strategy, a Monte Carlo simulation is performed with a variety of aerodynamic coefficient deviations and atmospheric density deviations that are subject to a Gaussian distribution given in Table 1. The reference trajectory is planned by the trajectory planning algorithm of [27] in an ideal environment.

**Figure 3:**
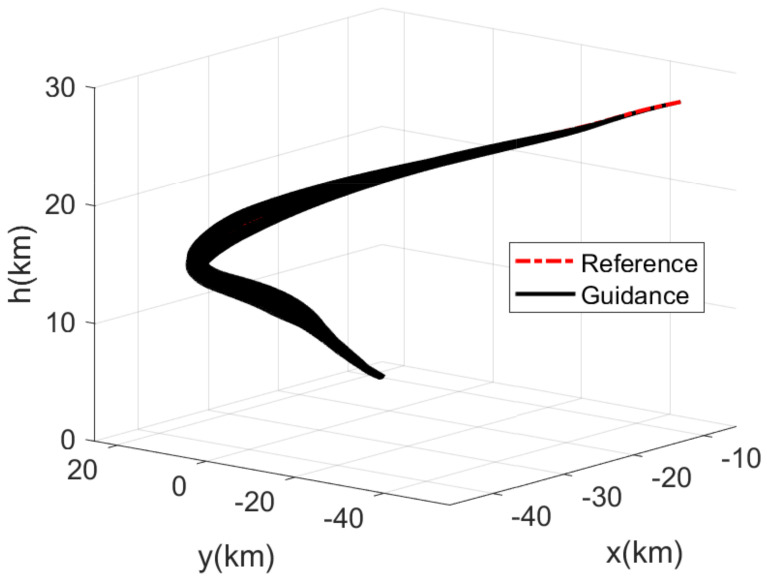
Three-dimensional TAEM trajectories of RLV in Monte Carlo simulation.

**Figure 4:**
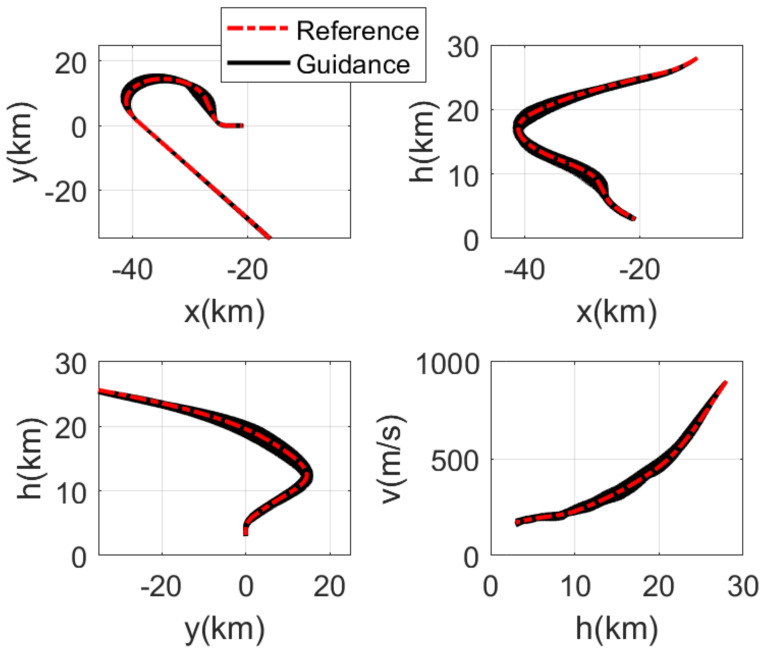
Three views of the TAEM trajectories and the velocity profiles with respect to altitude in Monte Carlo simulation.

**Figure 5:**
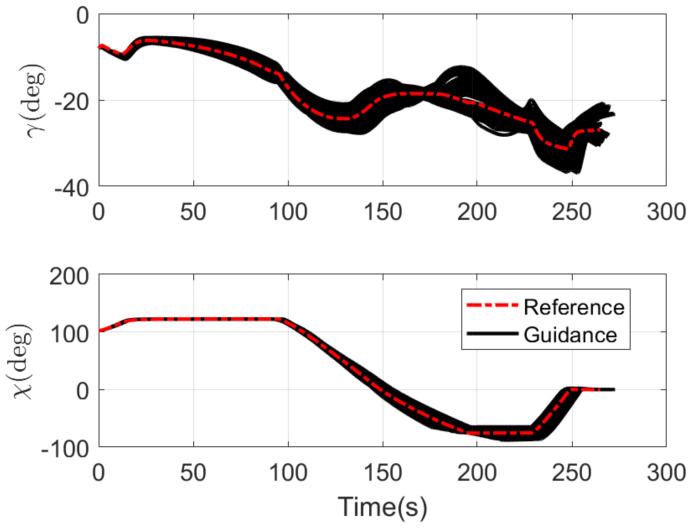
Flight path angle and heading angle profiles with respect to time in Monte Carlo simulation.

**Figure 6:**
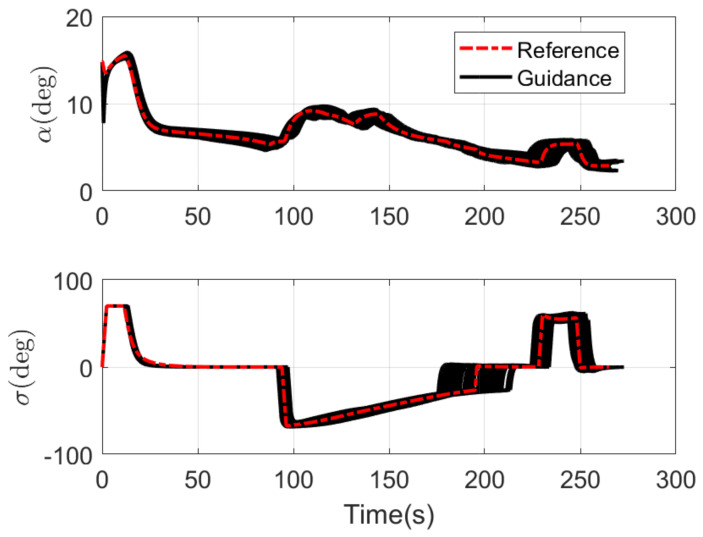
Angle of attack and bank angle profiles with respect to time in Monte Carlo simulation.

The RLV states of the reference trajectory and the 100 guidance trajectories are shown together in Figure 3, Figure 4, Figure 5 and Figure 6, where the dashed red line represents the reference trajectory, and the solid black lines represent the guidance trajectories. Figure 3 shows the three-dimensional TAEM trajectories of RLV in Monte Carlo simulation. The three views of the TAEM trajectories and the velocity profiles with respect to altitude in Monte Carlo simulation are depicted in Figure 4. Because the trajectory propagation simulation is terminated at the desired altitude h=3 km, the RLV can meet the terminal altitude constraints at ALI. The terminal velocities are all less than 180 m/s, meeting the requirements. The terminal errors of longitudinal position are within 0.3 km, and the terminal errors of lateral position are within 0.1 km. The flight path angle and heading angle profiles with respect to time in Monte Carlo simulation are shown in Figure 5. There is no strict requirement for terminal flight path angle in TAEM, thus angle errors are allowable. The terminal errors of heading angle are within 0.5 deg. Therefore, all the TAEM guidance trajectories of RLV meet the requirements of tracking accuracy. In addition, the angle of attack and bank angle profiles with respect to time in Monte Carlo simulation are illustrated in Figure 6. It can be seen that the guidance commands have been adjusted online to cope with the uncertainties and disturbances in the actual flight environment. The detailed terminal conditions of the 100 guidance trajectories are presented in Table 2, meeting all the terminal constraints. These Monte Carlo simulation results validate the effectiveness of the proposed intelligent guidance strategy.

### 4.2. Comparison Simulation

Under the influence of complex flight environment, RLV may deviate from the preset flight trajectory seriously and cannot return to the preset flight trajectory. To demonstrate the adaptability of the CACLA-based guidance strategy, deviations in initial conditions are applied to the RLV, where v=950 m/s, h=28.5 km, x=−5 km, y=−30 km. Then, a new reference trajectory is reconstructed by the trajectory planning algorithm in [27]. The parameters of CACLA are not changed, and a PID guidance law based on the preset reference trajectory is designed for comparison. In the guidance environment, the random aerodynamic coefficient deviation and atmospheric density deviation given in Table 1 are performed.

**Figure 7:**
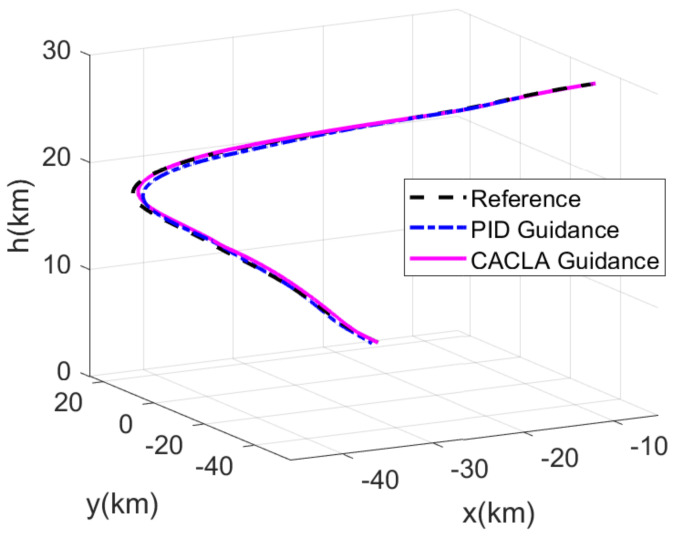
Three-dimensional TAEM trajectories of RLV in comparison simulation.

**Figure 8:**
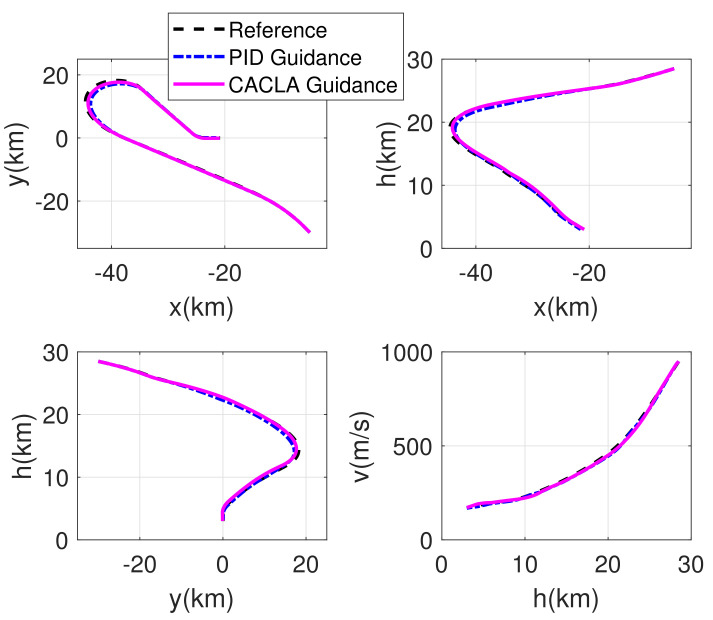
Three views of the TAEM trajectories and the velocity profiles with respect to altitude in comparison simulation.

**Figure 9:**
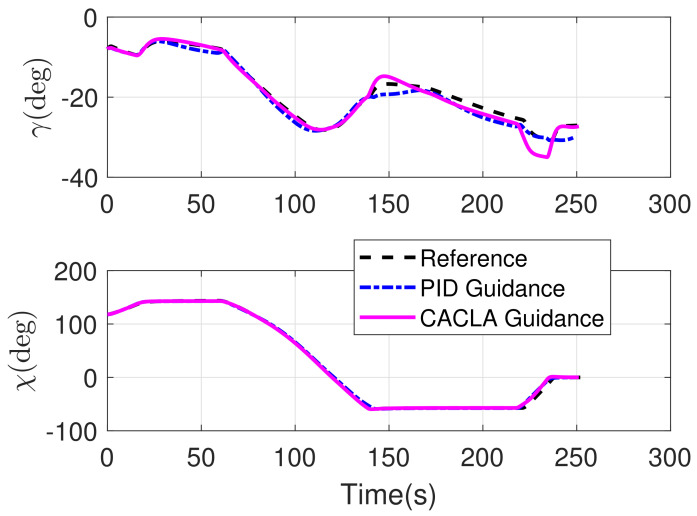
Flight path angle and heading angle profiles with respect to time in comparison simulation.

**Figure 10:**
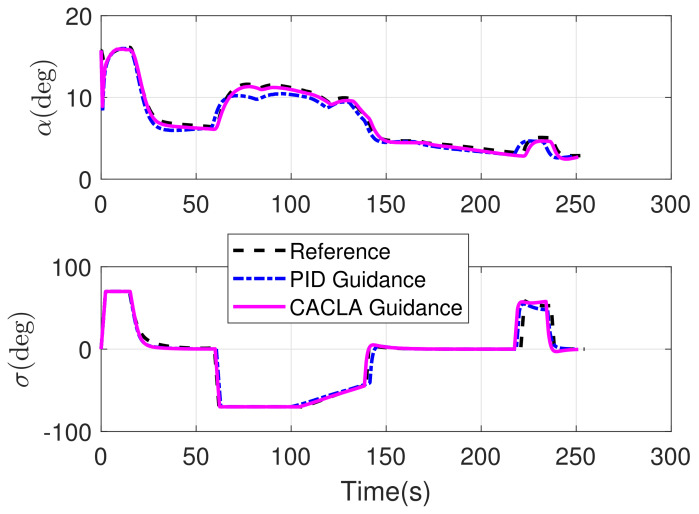
Angle of attack and bank angle profiles with respect to time in comparison simulation.

The RLV states of the reconstructed reference trajectory, the guidance trajectory by using CACLA-based guidance law and the guidance trajectory by using PID guidance law are shown together in Figure 7, Figure 8, Figure 9 and Figure 10. Figure 7 shows the three-dimensional TAEM trajectories of RLV in comparison simulation. The three views of the TAEM trajectories and the velocity profiles with respect to altitude in comparison simulation are depicted in Figure 8. Because the trajectory propagation simulation is terminated at the desired altitude h=3 km, the RLV can meet the terminal altitude constraints at ALI. The terminal velocities are less than 180 m/s, meeting the requirements. The terminal errors of lateral position are within 0.1 km. The terminal error of longitudinal position of CACLA guidance trajectory is within 0.3 km, meeting the requirements. However, the terminal longitudinal position of PID guidance trajectory is −21.5007, which does not meet the terminal error requirements. The flight path angle and heading angle profiles with respect to time in comparison simulation are shown in Figure 9. Although there is no strict requirement for terminal flight path angle in TAEM, the terminal flight path angle of CACLA guidance trajectory is closer to that of reconstructed reference trajectory than that of PID guidance trajectory. The terminal errors of heading angle are within 0.5 deg. In addition, the angle of attack and bank angle profiles with respect to time in comparison simulation are illustrated in Figure 10. The detailed terminal conditions of the reconstructed reference trajectory, the CACLA guidance trajectory and the PID guidance trajectory are presented in Table 3. It can be seen that the PID guidance law is inappropriate in the tracking task of the newly reconstructed reference trajectory. However, the CACLA-based guidance law can meet all the terminal constraints. Therefore, this comparison simulation results illustrate the advantages of the proposed CACLA-based guidance strategy.

## 5. Conclusions

This paper proposed an intelligent trajectory tracking guidance strategy for the TAEM phase of RLV. A reinforcement learning algorithm CACLA is applied to construct the guidance strategy of RLV, which has continuous state and action space. Two three-layer neuron networks are used to realize the function approximation for critic and actor, respectively. Then, an improved model-free TD learning algorithm is used in the weight updating process. A Gauss exploration is carried out to obtain the guidance commands of RLV. Finally, the Monte Carlo simulation and the comparison simulation have performed to show that the proposed guidance strategy can achieve the high-precision tracking of the TAEM reference trajectory with all ALI conditions satisfied. In addition, the CACLA-based guidance strategy is universal, and thus can be used not only in TAEM, but also in the initial re-entry phase and the automatic landing phase.

## Figures and Tables

**Table 1:** Model deviations for Monte Carlo simulations.

Parameter	Mean	Three Standard Deviations
Atmospheric density	0.0	15%
Aerodynamic lift coefficient	0.0	15%
Aerodynamic drag coefficient	0.0	15%

**Table 2:** TAEM terminal conditions in Monte Carlo simulation.

Conditions	Maximum	Minimum	Mean	Variance	Desired Value
$V_{f}$ (m/s)	178.8269	159.3518	167.9724	4.654	<180
$x_{f}$ (km)	−20.7174	−21.2804	−20.9724	0.15224	−21±0.3
$y_{f}$ (km)	0.0934	−0.0862	0.0043	0.0479	0±0.1
$\chi_{f}$ (deg)	0.1533	−0.0237	0.0638	0.0365	0±5

**Table 3:** TAEM terminal conditions in comparison simulation.

Conditions	Reference	CACLA Guidance	PID Guidance	Desired Value
$V_{f}$ (m/s)	169.9194	171.6748	168.3108	<180
$x_{f}$ (km)	−20.8309	−20.8800	−21.5007	−21±0.3
$y_{f}$ (km)	0.0148	−0.0863	0.0712	0±0.1
$\chi_{f}$ (deg)	0.0075	0.0828	−0.0414	0±5
